# In the Aftermath of Tragedies, Mass Graves Abound. Molecular Tools May Help Us Find Them.

**DOI:** 10.1021/acscentsci.5c01808

**Published:** 2025-10-06

**Authors:** Carolyn Wilke

## Abstract

With experimental burials, scientists are looking for chemical signposts that could help real-world investigations.

## Graphical abstract

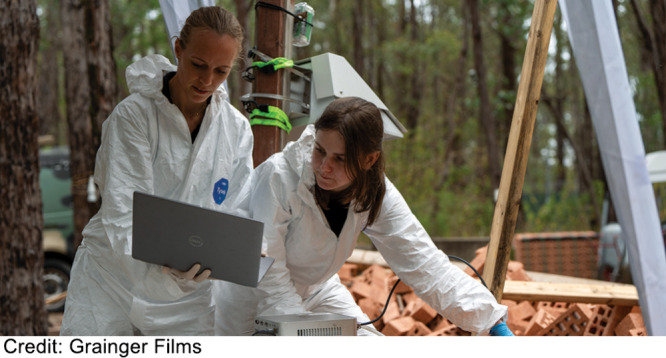

In May 2021, nine people were laid to rest in the sticky, clay-rich soil of San Marcos, Texas. It was not an ordinary burial: some of the individuals had been frozen for over a year. They were all clothed in the same blue T-shirts and shorts. These people had donated their bodies to science and were being interred in experimental graves at the Forensic Anthropology Research Facility’s body farm.

**Figure:**
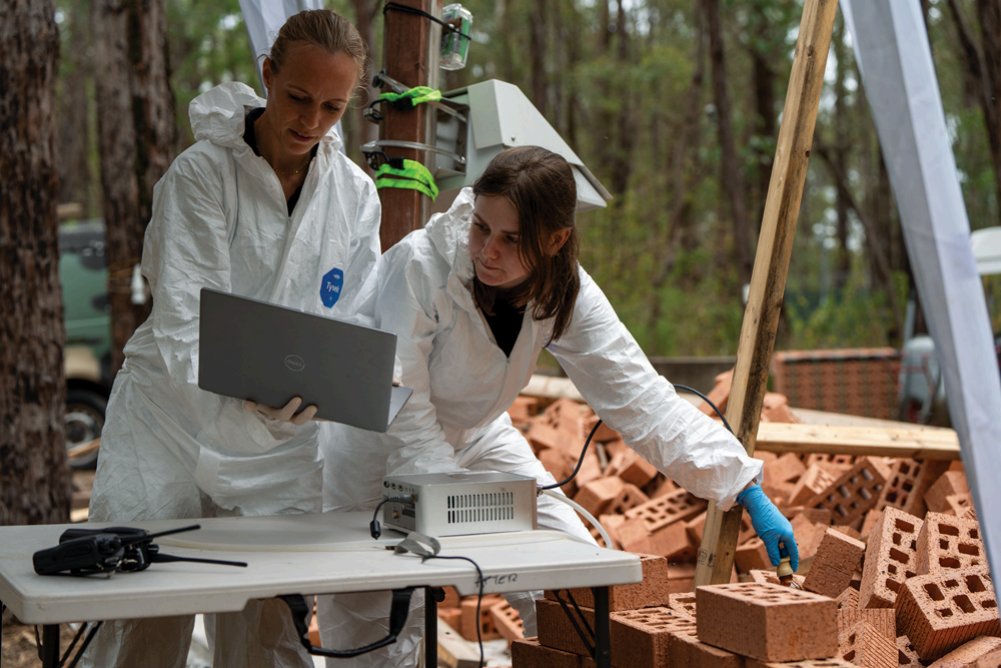
Forensic analytical chemist Maiken Ueland (left) and PhD candidate Bridget Thurn use an electronic nose to detect volatiles that may emanate from decomposing bodies. Credit: Grainger Films.

A research team buried six of the nine people in conditions meant to match small mass graves created during and after wars, genocides, and disasters. The researchers bound the wrists of several individuals and arranged the bodies atop one another. They wedged one body’s head against another’s knees or stomach. They placed some on their sides, others on their backs. Around the bodies, the researchers scattered coins, wallets, and jewelry—personal effects typically found in a hastily dug mass grave. Though the placement of the cadavers looked haphazard, “they were actually placed in a gentle way,” says Noemi Procopio, a biotechnologist at the University of Lancashire.

The six bodies would remain there for 18 months, providing snapshots of the physical and chemical processes that occur when many people are buried together. Researchers buried the other three people in nearby individual graves for comparison.

“The way bodies decompose in mass graves is very different from how a single body would decompose,” says Hayley Mickleburgh, an archeologist at the University of Amsterdam who is leading the Mass Grave Project at San Marcos. The environment and how perpetrators treated the bodies in criminal mass graves can also influence the quality of evidence extracted from a grave. With an experimental grave, “we can observe all of these changing variables over time so that we can better predict what to expect in real graves.”

Through such simulations, researchers can study the use of geophysical techniques to locate graves and provide training in excavation. They are also capturing chemical data; scientists are tracking differences in decomposition between mass and individual graves and how these differences affect the soil environment and microbes. By analyzing the chemistry involved in the decomposition process, researchers hope to identify chemical signals that could one day be used to detect and investigate mass graves.

Mickleburgh hopes to find a baseline profile for what happens in a mass grave that can be used to support actual investigations. For instance, chemistry and biochemical markers might provide insights into a grave’s timeline. “If we can be more precise,” she says, “we can pinpoint the time when a grave was created and the subsequent times when new bodies were added.” That could be useful when a legal defense claims that a grave predates an event in question.

## Preservation at the core

In 1950, British pathologist Arthur Keith Mant surveyed mass graves in the aftermath of World War II and documented how bodies decompose in them. Some of these sites contained hundreds, even thousands, of bodies, Mickleburgh says. In what he called the feather-edge effect, Mant described how bodies at the edge of a mass decomposed faster than those in the center. Even 3–5 years after burial, some bodies in the middle of a grave retained soft tissues. Investigators have observed similar patterns in other mass graves, such as those created during the genocide of Bosnian Muslims 30 years ago, Mickleburgh says.

But information from the investigations of atrocities is often protected and not published, Mickleburgh says. So researchers have created a few experimental graves that have reproduced the patterns that Mant saw. Bodies at the core of even a small experimental grave with six individuals can retain preserved soft tissue—including identifying marks such as tattoos and scars, Mickleburgh says.

“Even in some cases, we have the ridge detail on the fingertips,” Mickleburgh says.

Depending on their placement, bodies experience differences in exposure to oxygen and water, temperature, and the collection of microbes nearby. In an experimental grave at the University of Tennessee, Knoxville’s Anthropology Research Facility, three individuals were stacked in clay-rich soil for four years. The grave’s bottom became a bathtub-like basin filled with rainwater, says Jennifer DeBruyn, an environmental microbiologist at UT Knoxville. When researchers examined the grave, they found water pooled in the bottom. A hardly decomposed cadaver sat in this liquid, which was surrounded by clayey soil with low oxygen permeability. But from the top cadaver which experienced an aerated environment, only bones remained

## Signatures and signposts

While environmental differences influence how bodies degrade, bodies change their surrounding environments as they decompose. In individual graves, DeBruyn’s team has observed that the initial decomposition flushes the soil with ammonium, which is released as the body’s proteins break down. Close to a cadaver, salinity and ammonium levels become too intense for plants, which die off. But the release of nitrogen and carbon boosts the activity of some bacteria, which use up the soil’s oxygen. Meanwhile, the decomposition releases fluids and fats into the soil that prevent oxygen from diffusing in as quickly as microbes use it. The temporary low-oxygen state halts the aerobic process that converts ammonium to nitrates.

**Figure:**
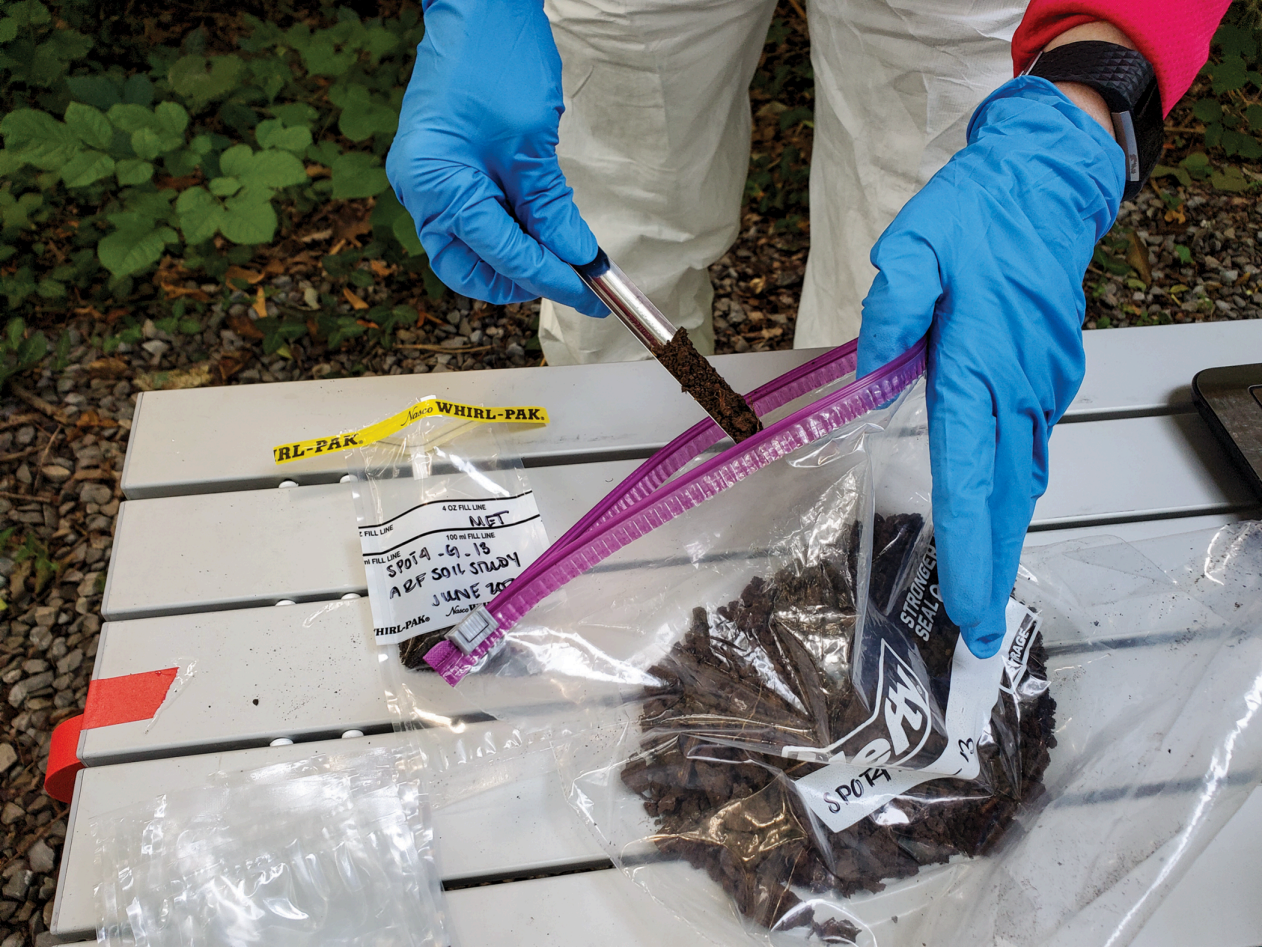
Mallari Starrett samples soil at the Anthropology Research Facility at the University of Tennessee, Knoxville, to learn about how decomposition changes soil geochemistry. Credit: Jennifer DeBruyn.

The bigger the body, the bigger the ammonium pulse, DeBruyn says. With a mass grave, “theoretically that would create a longer and bigger period of hypoxia.”

In addition to increasing nitrogen levels, decomposing bodies alter levels of other elements in the soil. Decomposition releases phosphorus, sulfur, and potassium. DeBruyn’s team measured elevated levels of positively charged ions, including manganese, magnesium, calcium, selenium, and boron, from the soil. And last, an uptick in metals, such as iron, copper, and zinc, likely occurs because of a drop in pH near the bodies.

While researchers have observed mostly the same chemical changes from buried humans and animals, the drop in pH seems unique to humans. With all the animals they’ve studied so far—including pigs, beavers, alligators, rabbits, mice, and tortoises—researchers from DeBruyn’s team have measured an uptick in pH.

DeBruyn says she doesn’t know what causes this difference. One idea is that human decay releases large amounts of fatty acids. But soil pH can be highly local. Using pH to detect mass graves or prove that a grave once existed in a certain location is “not really feasible,” Mickleburgh says. Her team found that one of the graves they studied had a spot of low pH thanks to the formic acid from fire ants that made a nest there.

Plants, too, may be helpful for indicating grave sites after enough time has passed for the environment to become hospitable to them again. In a study of two mass graves, one containing six individuals and another containing three, researchers saw that grave sites become a nutrient source. “We’ve seen particular types of grasses and plants that may grow on graves or near remains that we don’t see in the rest of the environment,” says forensic analytical chemist Maiken Ueland, director of the Australian Facility for Taphonomic Experimental Research at the University of Technology Sydney.

Plants and soil could also herald a decomposition event with clues invisible to human eyes. Plants produce chemicals in response to myriad environmental changes. A flood of nutrients and elements may be reflected in light bouncing off of a plant—or even soil—and detected through a technique called hyperspectral imaging. While this method may not distinguish between human and animal decomposition, it could allow investigators to identify sites for further investigation, and it can be deployed remotely by drones.

Mass graves may also be marked by smell. Decomposing bodies release volatile organic compounds, including aldehydes, ketones, hydrocarbons, and smelly sulfurous chemicals. Ueland’s team is prototyping electronic noses to detect these characteristic chemicals. Though odors would be strongest during a body’s active decomposition, volatiles could be detected even after bodies have become skeletons, she says.

Electronic noses could help in complicated disaster or mass grave environments where it’d be difficult to deploy scent-sniffing dogs, Ueland says. “If it’s dangerous to send a human to search, it’s also dangerous to send a dog to search.”

## Biomolecular clues

After investigators locate a mass grave, they work to identify those buried. Traditionally, they acquire genetic material from hard tissues, such as bones and teeth, Mickleburgh says. But processing those samples can be costly and requires time-consuming protocols. Soft tissues or swabbed samples would be easier to obtain and process. Before Mickleburgh’s team began its research, it wasn’t clear how reliable these samples would be and whether the quality of DNA preservation varies at different parts of a grave.

When the bodies in the Mass Grave Project were exhumed after 18 months, members of Mickleburgh and Procopio’s team swabbed relatively accessible areas, including the rectum, mouth, and eye sockets. Those in the mass grave had better-preserved DNA than those in the individual graves. As expected, the DNA of individuals in the mass grave was more degraded than it had been before burial. But the team was able to recover enough DNA from all three swabbed areas to match samples against those collected before burial.

Time since death can be another important clue for investigators. Procopio studies how certain types of molecules can serve as molecular timers. For mass and single graves, “there are striking differences also at the level of proteins, metabolites, and lipids,” Procopio says. The timing predicted by bone proteins from the project’s mass grave didn’t fit with the models she had previously developed based on individual burials.

A deceased person’s clothing may influence what biomolecules are detected. Natural textiles tend to trap decomposition fluid, the by- and end products of the body’s breakdown, Ueland says. Ueland’s team has extracted lipids, particularly sterols and fatty acids, from textiles. These molecules can help create a decomposition timeline.

**Figure:**
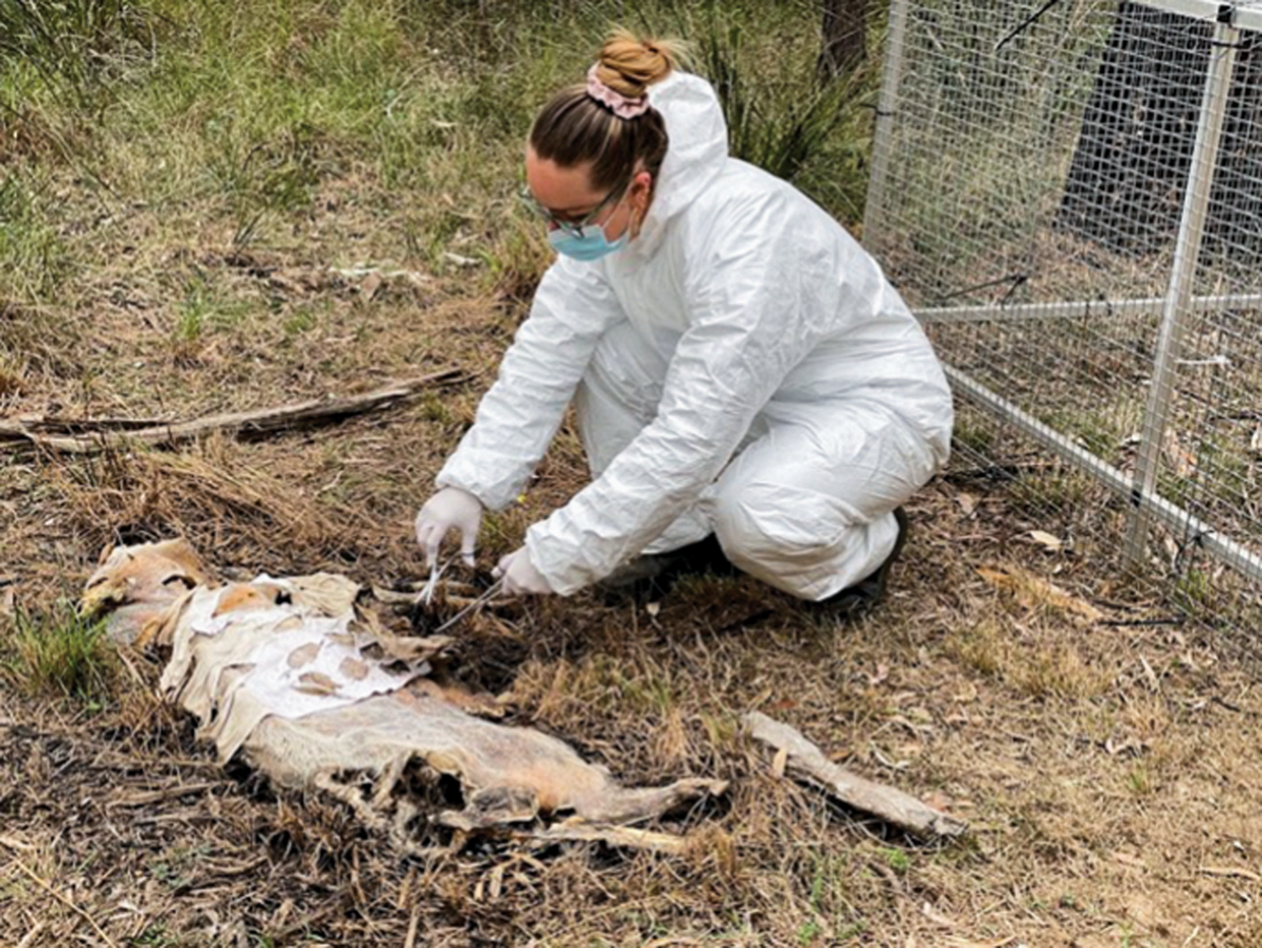
Researcher Sharni Collins samples textiles, which collect lipids, from a decomposing pig to compare with textile samples from human bodies. Credit: Australian Facility for Taphonomic Experimental Research.

Using multiple methods together may allow researchers to better gauge time since death in complicated mass grave environments. Metabolites survive for the least amount of time and may be useful only in the short term, while proteins and lipids last longer, Procopio says. Based on results from individual graves, the microbiome in the surrounding soil may be able to reveal the time of death to within a few months, even if a body has been buried for a long time. “If you remove a body after years, you will still have evidence of the fact that decomposition took place in that soil,” she says.

## Applying the knowledge

After 18 months, Mass Grave Project researchers exhumed the donors they’d buried in San Marcos, and the biochemical aspect of the project mostly came to an end. “It was nice to find, finally, some molecular evidence of what is being observed for years and years, which is this differential decomposition" between mass and individual graves, Procopio says. The project’s researchers had also collected a wealth of other data. For instance, after monitoring their grave sites with geophysical methods, they found that electrical resistivity and ground-penetrating radar could be used to identify areas that might contain a mass grave. They also used drones to track the site’s thermal signature, observing that the mass grave’s heat signal is most apparent before dawn. Such findings could help guide how investigators search for mass graves

While researchers have not come away with a single chemical method for locating mass graves or identifying those buried, the work has offered starting points for many potential techniques. They are still looking for insights that different biomolecules can provide: for instance, whether recovered DNA could allow to them work out physical characteristics—eye, hair, or skin color—of buried individuals, or whether isotopes could reveal where a buried person once lived.

After the initial 18-month burial, Mickleburgh’s team created what’s called a secondary mass grave. This time, Mickleburgh separated limbs from bodies, detached hands and feet, and buried the individuals again, simulating what happens when bodies are unearthed and moved.

Mickleburgh and colleagues exhumed the bodies from the secondary site after another 12 months. In this last excavation, Mickleburgh saw those individuals—whom she had once seen frozen in their newly deceased condition—as skeletal remains. For her, the experience reinforced their humanity. “They had lives and experiences,” she says. “That’s something that we should never forget—whether we’re looking at a 5,000-year-old grave or one that dates to yesterday.”

*Carolyn Wilke is a freelance contributor to* Chemical & Engineering News *, an independent news publication of the American Chemical Society.*

